# MicroRNA-phytochemical interactome targeting thioredoxin-interacting protein: a narrative review at the nexus of RNA therapeutics, cancer biology, and metabolic regulation

**DOI:** 10.3389/fonc.2026.1907571

**Published:** 2026-08-25

**Authors:** Peter Chinedu Agu, Patrick Maduabuchi Aja, Cecilia Ogechi Ofor, Kehinde Ross, Jun Lu

**Affiliations:** 1 Key Laboratory of Luminescence Analysis and Molecular Sensing, Ministry of Education, College of Pharmaceutical Sciences and Chinese Medicine, Southwest University, Chongqing, China; 2 Department of Biochemistry, College of Science, Evangel University, Akaeze, Nigeria; 3 Department of Biochemistry, Faculty of Biomedical Sciences, Kampala International University, Busenyi, Uganda; 4 Centre for Artificial Intelligence and Multidisciplinary Innovations Studies, Department of Auditing, College of Accounting Science, University of South Africa, Pretoria, South Africa; 5 Department of Biochemistry, Faculty of Science, Ebonyi State University, Abakaliki, Nigeria; 6 School of Pharmacy and Biomolecular Science, Liverpool John Moores University, Liverpool, United Kingdom; 7 Institute for Health Research, Liverpool John Moores University, Liverpool, United Kingdom

**Keywords:** cancer biology, microRNA, molecular docking, oxidative stress, phytochemicals, RNA therapeutics, RNA–small molecule interactions, thioredoxin-interacting protein (TXNIP)

## Abstract

MicroRNAs (miRNAs) are increasingly recognized as central regulators of gene expression, cellular adaptation, and disease progression. This is fundamentally reshaping current understanding of disease molecular pathogenesis and therapeutic intervention. Beyond their established roles in development and metabolism, miRNAs actively participate in oncogenesis, metabolic dysfunction, inflammation, and redox homeostasis. Emerging evidence shows that phytochemicals can modulate miRNA-mediated regulatory networks by influencing miRNA biogenesis, expression, stability, and functional activity through transcriptional, epigenetic, and post-transcriptional mechanisms. Among these pathways, the thioredoxin-interacting protein (TXNIP) axis has attracted considerable attention because of its critical involvement in oxidative stress, inflammation, metabolic reprogramming, apoptosis, and cancer-associated signalling. For instance, dysregulated TXNIP expression is strongly associated with metabolic dysfunction-associated fatty liver disease (MAFLD), diabetes, cardiovascular diseases, neurodegenerative disorders, and multiple cancers, making it an attractive therapeutic target. This narrative review discussed emerging trends on phytochemical-mediated regulation of TXNIP-associated miRNAs, including miR-148b, miR-33a/b, miR-17-5p, miR-224, and miR-20a. Particular emphasis was placed on the conserved miRNA seed region as the principal determinant of target recognition, while discussing the emerging hypothesis that phytochemicals may allosterically modulate structurally accessible RNA motifs to influence miRNA conformation, stability, RNA-induced silencing complex loading, and target accessibility without disrupting canonical Watson-Crick base pairing. We further discussed molecular docking, RNA-specific molecular dynamics simulations, and complementary structural validation approaches as emerging tools for investigating RNA-ligand interactions. Therefore, this review has provided a mechanistic and translational framework integrating RNA biology, redox signalling, and precision medicine to guide future development of RNA-targeted phytochemical therapeutics for cancer, metabolic disorders, and other chronic diseases.

## 1 Introduction

Phytochemicals are naturally occurring bioactive compounds derived from fruits, vegetables, herbs, and medicinal plants that have attracted considerable attention because of their broad therapeutic potential in disease prevention and management. These plant-derived molecules exhibit diverse pharmacological activities, including antioxidant, anti-inflammatory, anticancer, and metabolic regulatory effects (1–3). Consequently, many phytochemicals have been recognized as nutraceuticals with promising applications in chronic disease prevention and precision healthcare (4). Unlike conventional synthetic drugs that often act on a single molecular target, phytochemicals typically exert pleiotropic biological effects by simultaneously influencing multiple signalling pathways, transcriptional regulators, and metabolic networks. Among these emerging mechanisms, modulation of non-coding RNA (ncRNA)-mediated gene regulation, particularly through microRNAs (miRNAs), has become an important area of investigation (5, 6). Increasing evidence has shown that phytochemicals can influence miRNA expression, maturation, and functional activity, thereby providing an additional molecular basis for their diverse biological effects (7). This expanding field has generated considerable interest in developing RNA-guided phytochemical strategies for managing complex diseases, including cancer and metabolic disorders (8).

MicroRNAs are short endogenous non-coding RNAs that regulate gene expression post-transcriptionally through complementary base pairing with target messenger RNAs (mRNAs), leading to translational repression or mRNA degradation (5, 9, 10). These regulatory molecules participate in numerous physiological processes, including cellular differentiation, apoptosis, immune regulation, oxidative stress responses, and metabolic homeostasis (11). Dysregulation of miRNA expression has been implicated in the pathogenesis of many chronic diseases characterized by inflammation, oxidative stress, and metabolic dysfunction (12). Rather than functioning as isolated regulators, miRNAs operate as central nodes within complex gene regulatory networks that integrate intracellular and environmental signals (13–15). Numerous studies have further demonstrated that phytochemicals influence the expression of disease-associated miRNAs by modulating transcriptional pathways, epigenetic mechanisms, redox signalling, and components of the miRNA biogenesis machinery (16, 17). Consequently, modulation of miRNA regulatory networks has emerged as a promising therapeutic strategy for diseases in which conventional pharmacological approaches have achieved only limited success.

Among the molecular pathways implicated in chronic disease, thioredoxin-interacting protein (TXNIP) has emerged as a central regulator of oxidative stress, inflammation, metabolic reprogramming, and apoptosis. TXNIP negatively regulates the thioredoxin (Trx) antioxidant system, thereby promoting reactive oxygen species accumulation and inflammatory signalling (18). Persistent TXNIP overexpression contributes to the development and progression of metabolic dysfunction-associated fatty liver disease (MAFLD), diabetes mellitus, cardiovascular diseases, neurodegenerative disorders, and multiple cancers (19, 20). Accordingly, suppression of aberrant TXNIP activity represents an attractive therapeutic strategy. Several miRNAs, including miR-148b, miR-33a/b, miR-17-5p, miR-224, and miR-20a, have been reported to regulate TXNIP expression by targeting its messenger RNA, thereby attenuating oxidative stress, inflammation, and metabolic dysfunction (21, 22). Understanding the molecular mechanisms governing these regulatory interactions may therefore facilitate the development of novel RNA-guided therapeutic approaches targeting TXNIP-associated disease pathways.

A defining feature of miRNA function is the highly conserved seed region, comprising approximately nucleotides 2–8 at the 5′ terminus, which mediates Watson-Crick base pairing with complementary sequences located within the 3′ untranslated region (3′UTR) of target mRNAs (23). Because this region largely determines target specificity, both computational (24, 25) and experimental (26, 27) studies have extensively investigated seed-mediated miRNA recognition. Advances in RNA structural biology have simultaneously revealed that mature miRNAs possess structurally accessible motifs—including hairpin loops, internal bulges, grooves, and flexible regions adjacent to the seed sequence—that may represent potential binding sites for RNA-targeted small molecules (28, 29). Rather than directly occupying the canonical seed nucleotides, which must remain accessible for target recognition, small molecules are increasingly proposed to modulate RNA function possibly through allosteric interactions that alter RNA conformation, stability, RNA-induced silencing complex (RISC) loading, or target accessibility. Although direct interactions between phytochemicals and TXNIP-regulating miRNAs remain largely hypothetical, emerging studies in RNA chemical biology suggest that such mechanisms are biologically plausible and warrant systematic computational and experimental investigation (30, 31).

Against this background, this narrative review examined the emerging interface between phytochemicals, miRNA biology, and TXNIP regulation in cancer and metabolic diseases. We x-rayed ongoing scientific explorations describing how phytochemicals influence TXNIP-associated miRNA regulatory networks through established mechanisms involving transcriptional regulation, epigenetic modulation, redox signalling, and miRNA biogenesis, while also presenting a conceptual framework for investigating potential RNA-ligand interactions using molecular docking, molecular dynamics simulations, and complementary structural and functional validation approaches. Therefore, rather than asserting established direct binding between phytochemicals and mature miRNAs, this review proposes a mechanistically grounded hypothesis for future investigation and enumerated an integrated roadmap for advancing RNA-guided phytochemical therapeutics in precision oncology and metabolic medicine.

## 2 TXNIP and its role in disease pathogenesis

TXNIP is a crucial regulator of cellular redox homeostasis and metabolic balance. As a key inhibitor of the Trx system, TXNIP plays a significant role in oxidative stress modulation, inflammatory responses, and energy metabolism (32, 33). It has been reportedly involved in multiple disease pathways, making it a potential target in MAFLD, diabetes, cardiovascular diseases, and neurodegenerative disorders (33). Additionally, TXNIP is highly responsive to metabolic and environmental stressors, including hyperglycemia, lipotoxicity, and oxidative damage, which trigger its overexpression and drive disease progression (34). Recent findings suggest that TXNIP expression can be modulated by non-coding RNAs, particularly miRNAs, which regulate its transcriptional and post-transcriptional activity (22, 35). Therefore, the involvement of TXNIP in disease pathogenesis highlights the necessity for targeted interventions that can modulate its expression, with miRNA-based regulatory mechanisms offering a promising approach.

### 2.1 TXNIP in oxidative stress and inflammation

**Figure 1:**
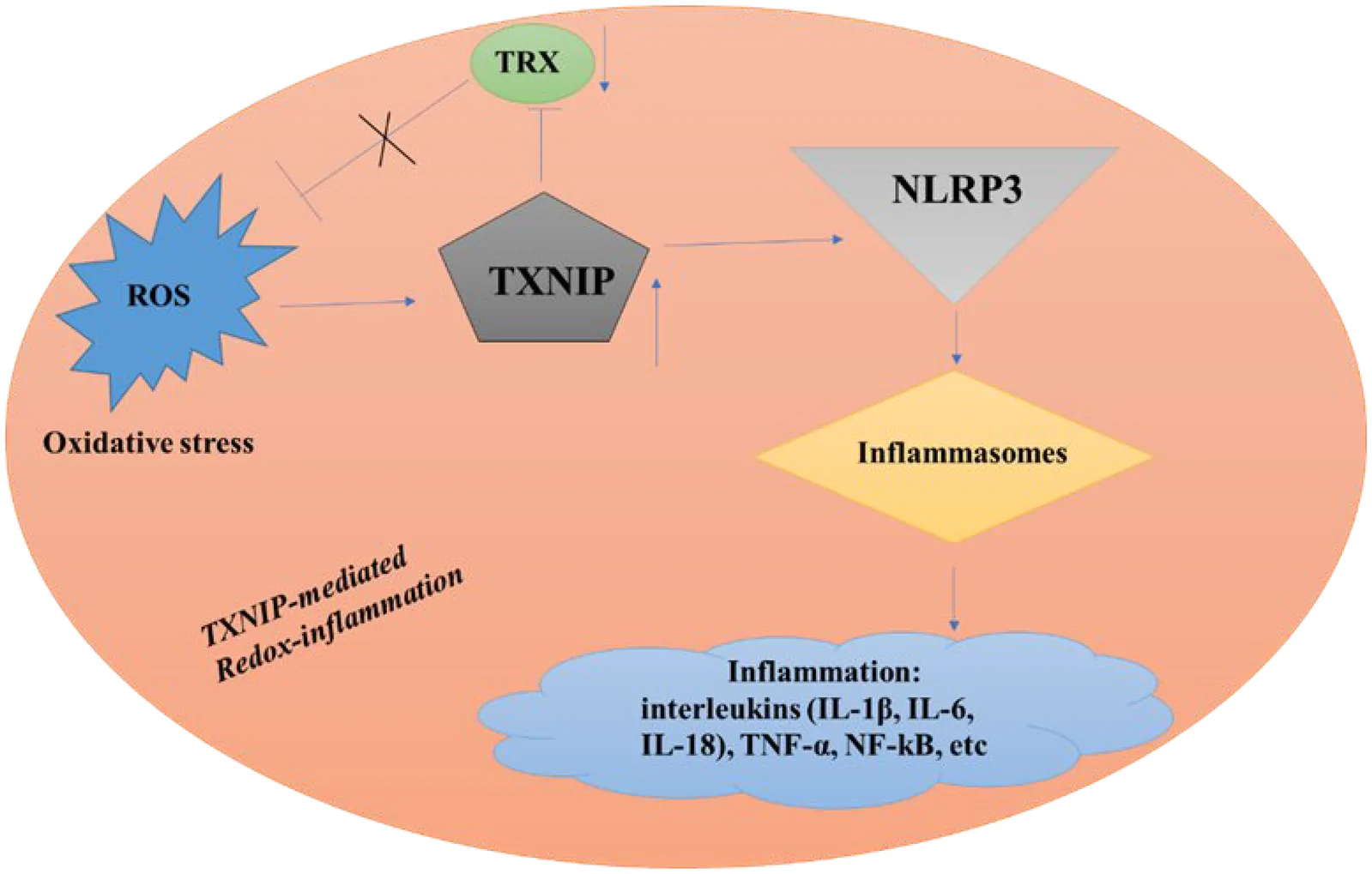
TXNIP-mediated redox-inflammatory pathway.

One of the main causes of cellular damage is oxidative stress, which is defined by an imbalance between the pro-oxidant and antioxidant systems and an excessive formation of reactive oxygen species (ROS). **Figure 1** represents TXNIP-mediated redox-inflammatory signaling.

According to Ji Cho et al. (36), TXNIP is a negative regulator of the thioredoxin system, a major antioxidant defense mechanism that scavenges ROS and maintains cellular redox homeostasis. Under physiological conditions, TXNIP serves as a metabolic switch that binds to Trx and inhibits it, thereby stabilizing its role in neutralizing oxidative stress (20). However, in disease states, TXNIP overexpression exacerbates ROS accumulation, leading to mitochondrial dysfunction, lipid peroxidation, and DNA damage (19). In addition to oxidative stress, TXNIP is a key mediator of inflammation. According to Yang et al. (37), it activates the nucleotide-binding oligomerization domain (NOD)-like receptor protein 3 (NLRP3) inflammasome, a multiprotein complex that triggers the release of pro-inflammatory cytokines such as tumor necrosis factor-alpha, nuclear factor kappa-B, interleukin-1β (IL-1β), and interleukin-18 (IL-18) (see **Figure 1**). This activation can contribute to chronic inflammation, a hallmark of metabolic disorders and age-related diseases (38). Consequently, TXNIP-driven inflammasome activation has been implicated in conditions such as non-alcoholic steatohepatitis (NASH) (39), insulin resistance (40), atherosclerosis (41, 42), prostatic disease (43), and Cerebrovascular and Neurodegenerative Diseases (44). Given its dual role in oxidative stress and inflammation, TXNIP has piqued scientific debate as a crucial therapeutic target for diseases where these mechanisms are central to pathogenesis.

### 2.2 TXNIP in metabolic disorders

**Figure 2:**
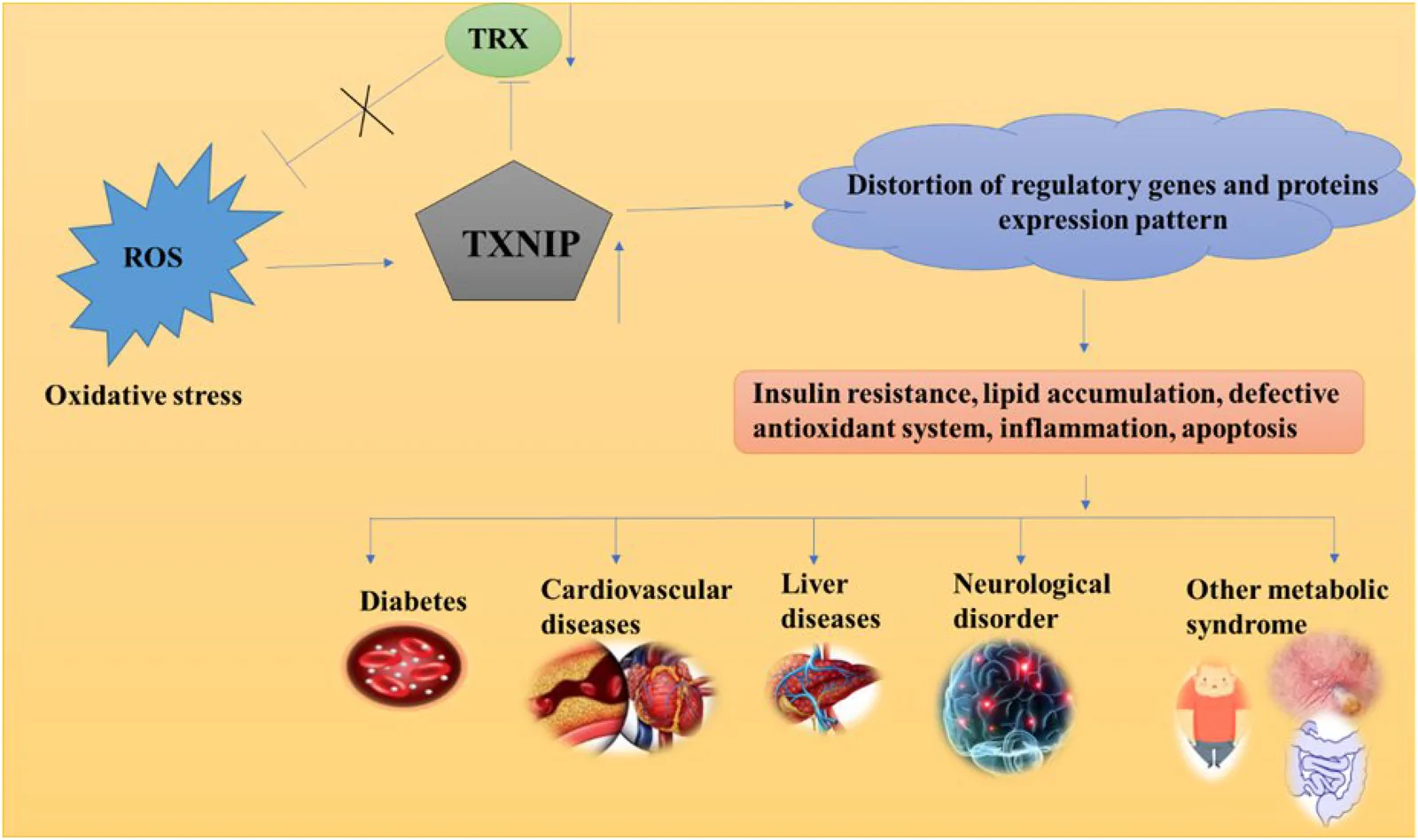
TXNIP in metabolic diseases.

TXNIP is widely recognized as a critical regulator of metabolic homeostasis, with its dysregulation contributing to the development of NAFLD (now MAFLD) and underlying pathophysiological conditions (44). In MAFLD, TXNIP overexpression is linked to hepatic insulin resistance, lipid accumulation, and increased susceptibility to liver fibrosis (39). According to studies, elevated TXNIP levels in hepatocytes enhance oxidative stress and inflammation (45). This exacerbates the transition from simple hepatic steatosis to more severe forms, such as NASH and cirrhosis, and even hepatocellular carcinoma. Similarly, an experimental study by Li et al. (46) has demonstrated that TXNIP knockdown or inhibition reduces hepatic lipid accumulation and improves mitochondrial function, underscoring its role as a metabolic regulator. A panoramic view of TXNIP involvement in disease pathogenesis is presented in **Figure 2**.

In diabetes, TXNIP is a major contributor to β-cell dysfunction and insulin resistance (45). It is highly upregulated in response to hyperglycemia, where it promotes pancreatic β-cell apoptosis and impairs insulin secretion (47). It has been proposed that TXNIP-induced oxidative stress and inflammation in pancreatic islets exacerbate the progression of type 2 diabetes to sustain hyperglycemia and metabolic instability (48). Wu et al. (49) also reported that TXNIP inhibits glucose transporter type 1 (GLUT1) and type 4 (GLUT4), thereby suppressing glucose uptake in insulin-sensitive tissues and contributing to further insulin resistance.

In cardiovascular diseases, TXNIP has been implicated in endothelial dysfunction, hypertension, and atherosclerosis. Li et al. (41) reported that TXNIP promotes vascular inflammation and oxidative stress to accelerate plaque formation and arterial stiffness. Additionally, TXNIP-mediated endothelial cell apoptosis and impaired nitric oxide signaling contribute to vascular complications and increase the risk of ischemic heart disease and stroke (42). Given its extensive role in metabolic dysregulation, targeting TXNIP can be a promising strategy for mitigating the progression of metabolic, neurological, and cardiovascular diseases.

## 3 MicroRNA and TXNIP regulation

**Figure 3:**
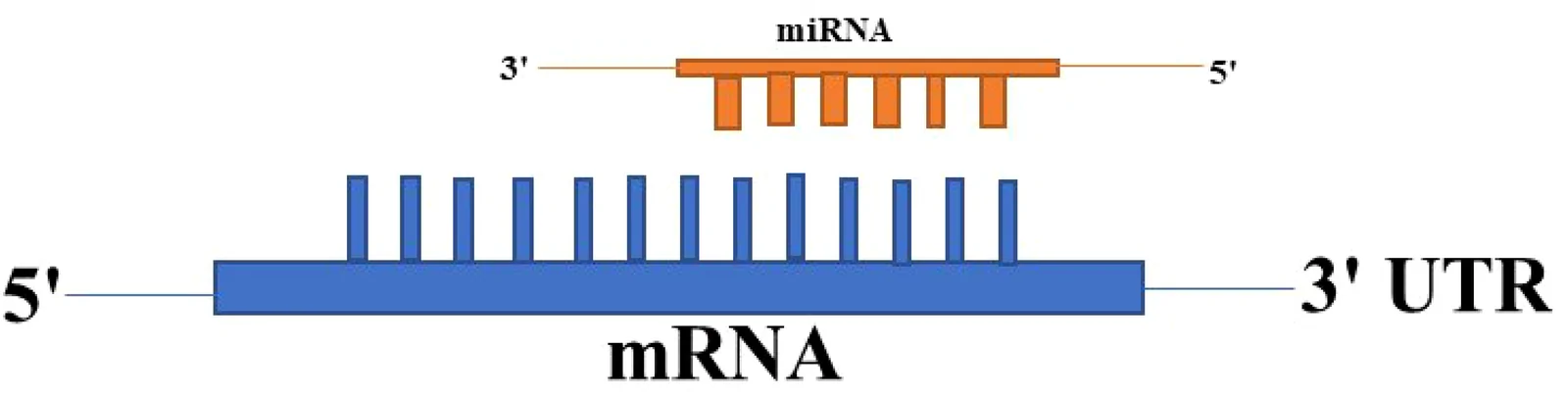
A typical miRNA-mRNA interaction.

As in **Figure 3**, miRNAs are small, ncRNA molecules that regulate gene expression post-transcriptionally by binding to the 3’ untranslated region (3’ UTR) of target mRNAs (5).

This interaction leads to translational repression or mRNA degradation, thereby modulating cellular processes such as oxidative stress, inflammation, and metabolic homeostasis (23). Interestingly, TXNIP, a crucial regulator of redox balance and metabolic function, is tightly controlled by several miRNAs (21). Reportedly, the dysregulation of TXNIP-targeting miRNAs has been implicated in metabolic disorders (50). Researchers are poised to understand the mechanisms through which miRNAs regulate TXNIP expression. This is to provide insights into novel therapeutic approaches.

### 3.1 miRNAs that target TXNIP

TXNIP expression can be tightly regulated at multiple levels. These levels involve transcriptional, post-transcriptional, and epigenetic mechanisms (51). Among these, miRNA-mediated post-transcriptional regulation has emerged as a critical pathway for controlling TXNIP expression in health and disease (22). Consequently, several miRNAs have been identified as negative regulators of TXNIP that play a protective role against oxidative stress and metabolic disorders. Notable examples are miR-148b, miR-33a/b, miR-17-5p, miR-224, and miR-20a.

- miR-17-5p: This miRNA downregulates TXNIP expression to reduce oxidative stress and inflammation. Studies have demonstrated that miR-17-5p overexpression decreases TXNIP levels to improve insulin sensitivity and mitigate metabolic dysfunction (52).
- miR-33a/b: These miRNAs are involved in lipid metabolism and energy homeostasis. They inhibit TXNIP expression to improve glucose uptake and enhance mitochondrial function (53, 54).
- miR-148b: This miRNA has been shown to suppress TXNIP in hepatocytes, thereby reducing lipotoxicity and inflammation associated with fatty liver diseases (55).
- miR-224 and miR-20a: These miRNAs have been implicated as regulators of TXNIP under oxidative stress, particularly in diabetes and cardiovascular diseases (56).

Thus, TXNIP-mediated oxidative stress and inflammation may be reduced by therapeutic manipulation of these miRNAs, providing a viable approach to the treatment of metabolic diseases.

### 3.2 Seed region of miRNA

**Figure 4:**
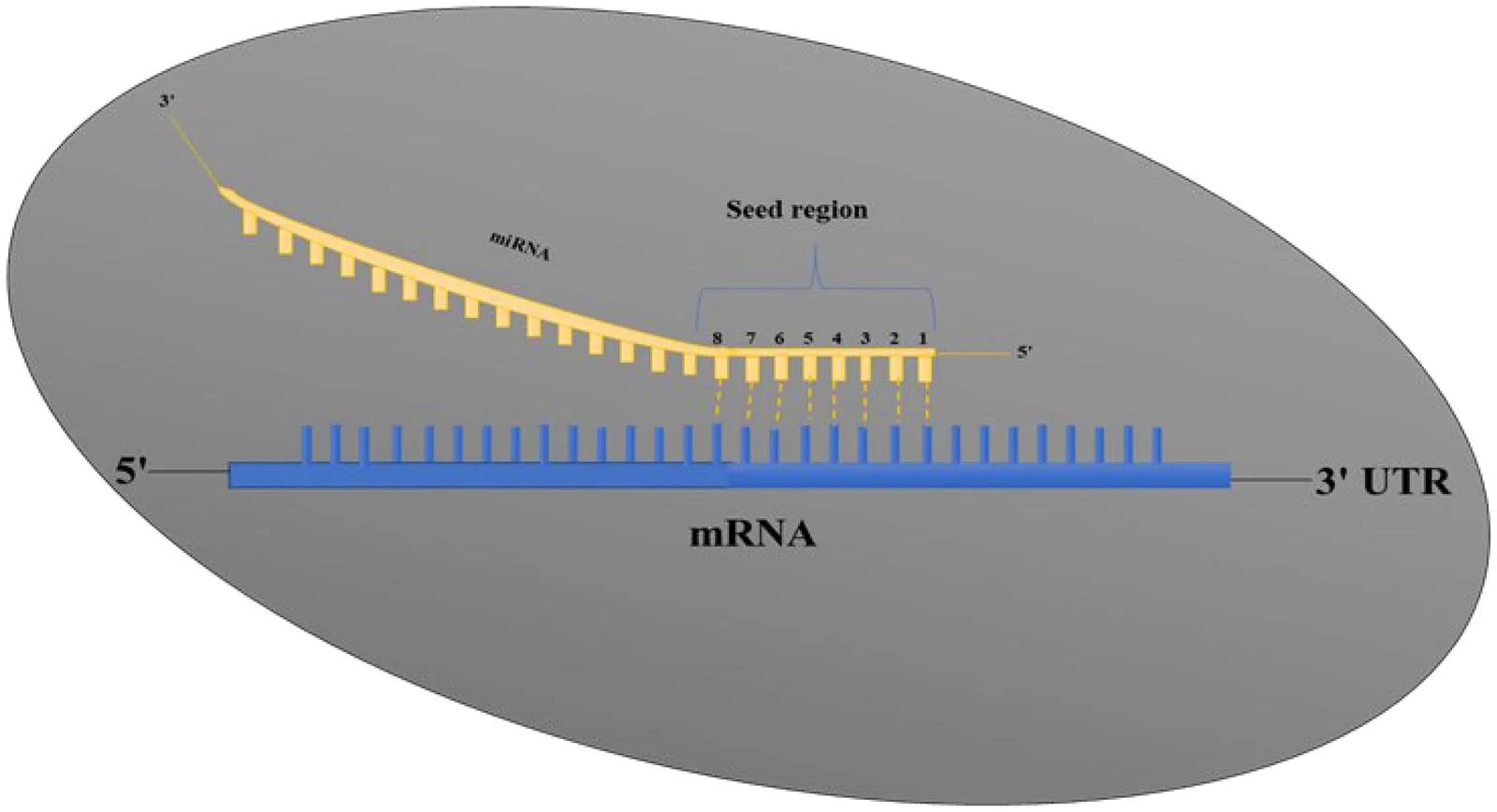
Schematic representation of the seed region.

As **Figure 4** schematically presents, the seed region of an miRNA is a short, highly conserved sequence of nucleotides located at the 5’ end of the mature miRNA molecule, typically spanning positions 2 to 8 as per Shin et al. (57).

Accordingly, this segment functions due to the ability of the miRNA to bind to complementary sequences within the 3’ UTR of target mRNAs. Furthermore, it is essential for the specific recognition of target mRNAs, and its interaction with the target sequence is a pivotal step in gene silencing. Therefore, due to this vital role of targeting mRNA, the seed region is considered a core element in determining the effectiveness and specificity of miRNA-mediated regulation (58).

#### 3.2.1 Important features of the seed region

The seed region is a crucial feature in miRNA biology, providing several important functions highlighted by Kehl et al. (23) and van der Kwast et al. (58).

- Primary binding site: The seed region is the main determinant for miRNA-mRNA interaction and establishes the initial binding interaction with the complementary sequence in the target mRNA’s 3’ UTR. Consequently, this interaction initiates downstream processes such as gene silencing or translation repression.
- Highly conserved: The seed sequence is typically highly conserved across different species and reflects the fundamental role in miRNA function. The conservation observed throughout evolution indicates that the miRNA seed region retains its functional consistency across different organisms, allowing miRNAs to regulate genes in a similar manner across various species.
- Regulates gene expression: The interaction between the seed region of the miRNA and its target mRNA leads to gene silencing. This regulation is primarily achieved by either mRNA degradation or translation inhibition, resulting in the downregulation of target gene expression.
- Determines target specificity: The seed region is responsible for the miRNAs’ target specificity. Even though miRNAs can regulate multiple genes, the seed sequence similarity between different target mRNAs is what allows a single miRNA to interact with and regulate many distinct genes. Therefore, this feature contributes to the multifaceted roles of miRNAs in various biological processes, including development, differentiation, and disease regulation.

#### 3.2.2 Seed region types and variations

According to Kehl et al. (23), the intensity and kind of the interactions vary depending on the precise sequence and length of the seed region, even though the standard seed region generally refers to positions 2–8 of the miRNA. This distinguishes canonical from non-canonical seed region binding.

##### 3.2.2.1 Canonical seed region binding

The canonical regions are classified into three, highlighted per Grimson et al. (59).

- 6-mer (positions 2-7): The weakest form of seed interaction. Although it can still lead to target regulation, the binding is less stable, and the effect is often weaker.
- 7-mer (positions 2-8): This is a more stable and common seed region interaction characterized by stronger miRNA-mRNA binding and more efficient gene silencing.
- 8-mer (positions 2–8 with Adenine at position 1): The strongest and most stable seed-region interaction. Here, the Adenine at position 1 enhances binding strength and specificity and initiates more effective gene regulation.

##### 3.2.2.2 Non-canonical or off-seed region binding

In some cases, miRNAs can still regulate gene expression without strict complementarity to the canonical seed region (60). These non-canonical interactions may involve additional binding sites outside the seed region, leading to partial or weak binding. This kind of regulation, although rare, demonstrates the adaptability of gene silencing mediated by miRNA and the possibility of miRNA-mRNA interactions that do not adhere strictly to conventional seed-pairing rules (61, 62).

Nevertheless, the miRNA seed region is a crucial element in regulating gene expression. By interacting with complementary target sequences in the 3’ UTR of mRNAs, the seed region governs the specificity and efficacy of miRNA-mediated silencing (63). Conversely, variations in the length and complementarity of the seed region further enhance the adaptability of miRNA action, allowing for nuanced control of gene expression in both health and disease.

### 3.3 Mechanism of miRNA target recognition

The recognition of target mRNAs by miRNAs is a highly defined process, with the seed region being key to binding in different ways. The mechanisms are hereby discussed according to Bartel (64).

- Base-pairing interaction: The seed region of a miRNA typically forms a Watson-Crick base-pairing interaction with a complementary sequence in the 3’ UTR of a target mRNA. This interaction is highly definite and is often the initial and most important step in miRNA-mediated regulation.
- Perfect or near-perfect binding: When the seed region and the target mRNA sequence have perfect or near-perfect complementarity, the Argonaute protein (part of the RNA-induced silencing complex or RISC) facilitates the degradation of the target mRNA. This leads to mRNA cleavage and a reduction in the expression of the targeted gene. Importantly, the degree of complementarity between the miRNA seed region and its target mRNA largely determines the fate of the target mRNA.
- Imperfect binding: The seed region may bind imperfectly to its complementary target sequence and cause translation repression rather than mRNA degradation. Phenomenally, this occurs when the binding affinity is weaker, and its results cause inhibition of protein synthesis without complete mRNA degradation. Noteworthily, this mechanism allows for more fine-tuned regulation of gene expression.

**Figure 5:**
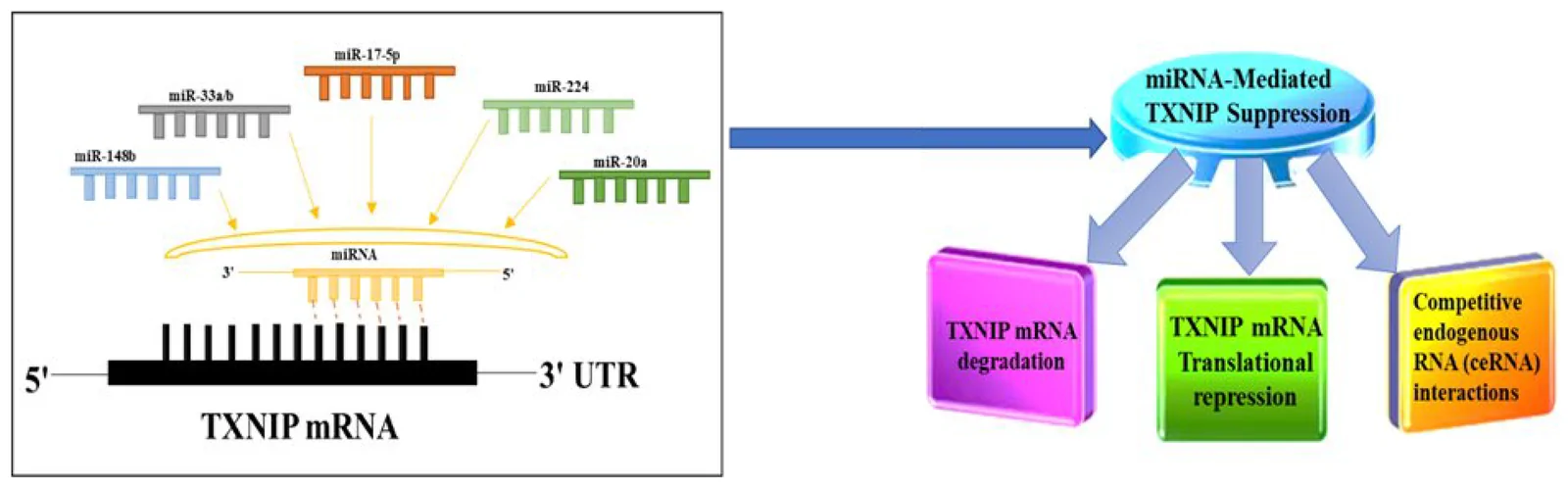
Primary mechanisms of miRNA-mediated TXNIP suppression.

Furthermore, the suppression of TXNIP by miRNAs occurs through multiple mechanisms. In **Figure 5**, four ways are primarily involved and were highlighted with the insight from Chen et al. (65).

- mRNA degradation: This is when miRNAs recruit argonaute proteins to form the RNA-induced silencing complex (RISC) that leads to TXNIP mRNA degradation. It results in a significant reduction in TXNIP protein levels and can alleviate oxidative stress and inflammatory responses.
- Translational repression: Sometimes, instead of degrading TXNIP mRNA, certain miRNAs inhibit its translation by preventing ribosome assembly. This mechanism allows for the dynamic regulation of TXNIP levels in response to cellular stressors (6).
- Competitive endogenous RNA (ceRNA) interactions: Other non-coding RNAs, such as long non-coding RNAs (lncRNAs) or circular RNAs (circRNAs), can act as miRNA sponges, sequestering TXNIP-targeting miRNAs and reducing their inhibitory effects. Consequently, it disrupts these interactions, which could have enhanced miRNA-mediated TXNIP suppression.

## 4 Phytochemicals as modulators of miRNA activity

The wide-ranging regulatory effects of phytochemicals, which are naturally occurring bioactive substances produced from plants, on cellular signalling pathways have garnered growing interest. Modulation of miRNA-mediated gene regulation has been identified as one of the key mechanisms behind their therapeutic potential. miRNAs are essential post-transcriptional regulators of gene expression, and there is mounting evidence that phytochemicals may affect miRNA synthesis, stability, and functional activity either directly or indirectly (7). Through these regulatory actions, phytochemicals can fine-tune critical pathogenic pathways, including those involving TXNIP, oxidative stress, inflammation, and metabolic dysregulation. Mutually, this establishes phytochemicals as prospective modulators of RNA regulatory networks important to metabolic syndrome and cancer-related illnesses.

### 4.1 Potential mechanisms of phytochemicals’ influence on miRNA activity

**Figure 6:**
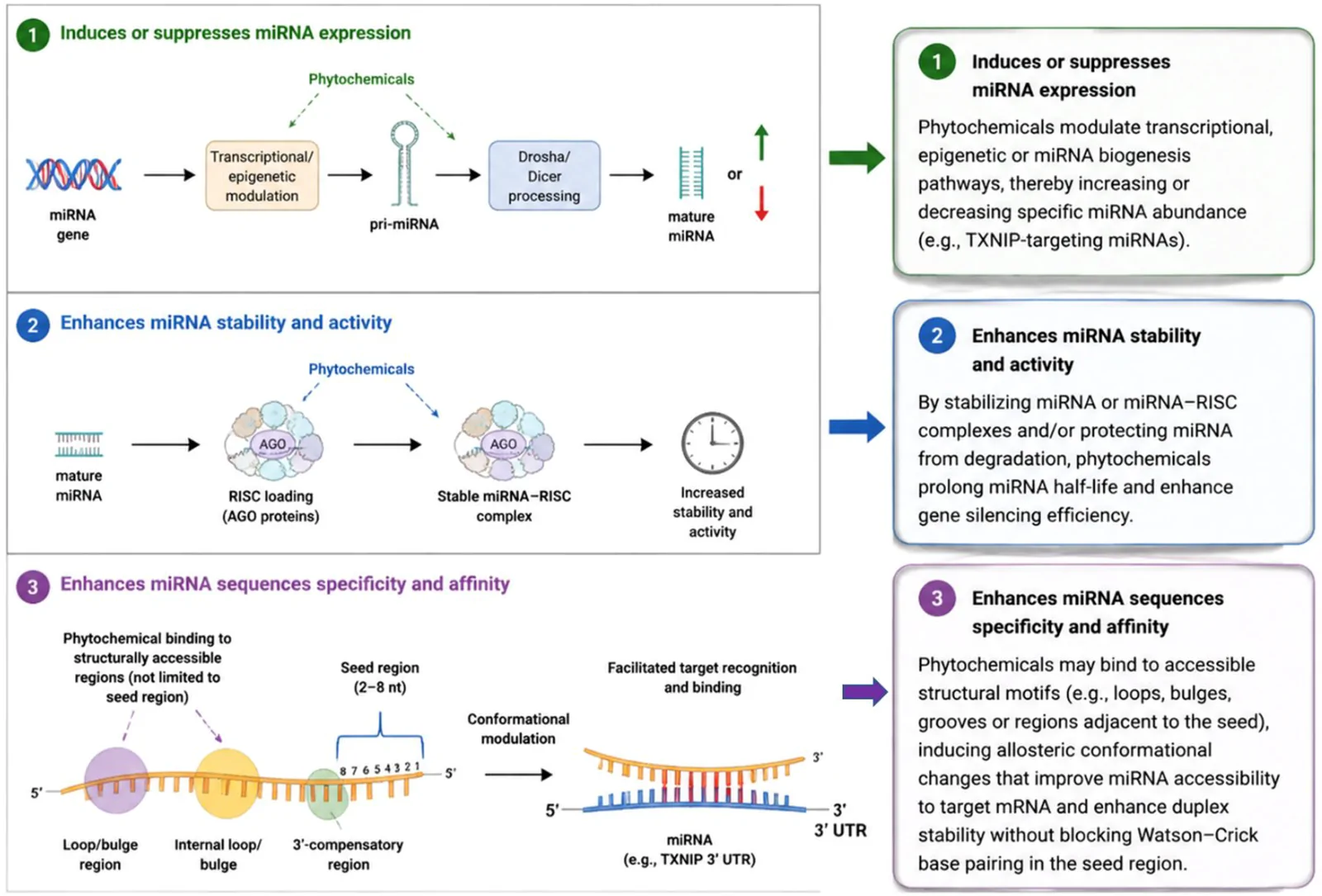
Potential mechanisms of phytochemicals’ influence on miRNA activity.

Instead of relying solely on sequence-specific genetic targeting, phytochemicals have been suggested to affect miRNA activity through a variety of indirect and RNA-contextually-dependent ways. These processes are based on well-established RNA biology principles as well as newly discovered RNA–small-molecule interactions (30, 66). According to this conceptual framework, non-covalent interactions involving hydrogen bonding, hydrophobic contacts, and electrostatic forces between functional groups of phytochemicals, RNA nucleotides, and the phosphate backbone can affect miRNA maturation, RISC loading, structural stability, or RNA accessibility (31, 67). **Figure 6** shows a schematic illustration of these potential interactions. However, these mechanisms are hypothesized as models that need more experimental and computational confirmation.

#### 4.1.1 Phytochemicals as inducers or suppressors of miRNA expression

Phytochemicals may regulate miRNA abundance indirectly by modulating upstream transcriptional or post-transcriptional regulatory pathways rather than directly altering miRNA sequences. For instance, they can influence transcription factors, epigenetic regulators, or enzymes involved in miRNA biogenesis (such as Drosha and Dicer), thereby altering the maturation efficiency of specific miRNAs. Through these pathways, phytochemicals can either enhance or suppress the levels of miRNAs implicated in TXNIP regulation. Increased expression of TXNIP-targeting miRNAs can subsequently contribute to reduced TXNIP expression and attenuation of downstream oxidative and inflammatory signaling pathways (16, 67). Thus, this indirect regulatory axis represents a plausible mechanism for phytochemical-mediated RNA modulation in disease situations.

#### 4.1.2 Phytochemicals as enhancers of miRNA stability and activity

Phytochemicals may also influence miRNA function by modulating the stability of miRNA-protein complexes, particularly the RNA-induced silencing complex (RISC). By stabilizing miRNA incorporation into AGO-containing effector complexes or reducing RNA degradation pathways, phytochemicals can prolong miRNA half-life and enhance post-transcriptional gene silencing efficiency. Such stabilization effects could amplify the regulatory impact of miRNAs targeting TXNIP and other stress-response genes (68). In this situation, phytochemicals can increase the functional lifetime of miRNA-mediated gene suppression by acting as modulators of RNA-protein interaction dynamics rather than direct sequence-specific binders of mature miRNAs.

#### 4.1.3 Phytochemicals as modulators of miRNA structural accessibility and target recognition

Emerging evidence in RNA chemical biology suggests that small molecules can interact with RNA structures through non-covalent binding to accessible motifs such as internal loops, bulges, hairpin regions, and phosphate backbones (31, 66). In this framework, phytochemicals may interact with structurally flexible regions of precursor or mature miRNAs, thereby inducing local conformational changes that influence miRNA accessibility and functional dynamics. Importantly, direct occupation of the canonical seed region (nucleotides 2–8), which is essential for Watson-Crick base pairing with target mRNAs, is biophysically unlikely to support enhanced miRNA-mRNA pairing. Instead, we suggest that phytochemical interactions are more plausibly associated with allosteric modulation of RNA structure, indirectly affecting seed accessibility, RISC loading efficiency, or duplex stability with target transcripts such as TXNIP mRNA. This model aligns more closely with established principles of RNA structural biology and emerging RNA–ligand interaction studies (30, 31).

These processes generally demonstrate the potential of phytochemicals as indirect, structural, and protein-mediated modulators of miRNA regulatory networks. They also promote more computational and experimental research into RNA-guided phytochemical therapies for metabolic and oncologic diseases and offer a physiologically rational framework for TXNIP modulation.

### 4.2 Phytochemical classes affecting miRNA expression

Phytochemicals are increasingly recognized as important modulators of RNA regulatory networks, capable of influencing miRNA expression and activity through multiple molecular mechanisms. Current evidence suggests that these compounds primarily regulate miRNA biogenesis, epigenetic modifications, transcriptional pathways, redox signaling, and RNA-processing machinery rather than directly interact with miRNA sequences. Consequently, phytochemicals may alter the abundance and functional activity of miRNAs involved in oxidative stress, inflammation, metabolism, and cancer progression. Among the numerous phytochemical classes investigated, flavonoids, polyphenols, and alkaloids have attracted particular attention because of their reported ability to regulate miRNAs associated with TXNIP expression and related disease pathways (69). Emerging advances in RNA chemical biology further raise the possibility that selected phytochemicals may allosterically modulate RNA structure or RNA–protein interactions, although these mechanisms remain largely hypothetical and require rigorous computational and experimental validation.

#### 4.2.1 Flavonoids

Flavonoids comprise a diverse group of naturally occurring polyphenolic compounds abundant in fruits, vegetables, tea, and numerous medicinal plants. Their antioxidant, anti-inflammatory, and cytoprotective properties have been extensively investigated in relation to chronic metabolic and inflammatory diseases. Increasing evidence indicates that flavonoids regulate the expression of several miRNAs involved in oxidative stress, inflammatory signaling, apoptosis, and cellular metabolism (70). Compounds such as quercetin and epigallocatechin gallate (EGCG) have been reported to influence miRNAs implicated in TXNIP regulation, thereby contributing indirectly to improved redox homeostasis and metabolic function (71). While the precise molecular mechanisms remain incompletely understood, these regulatory effects are thought to involve modulation of transcriptional signaling pathways, epigenetic regulation, and miRNA biogenesis rather than direct sequence-specific binding to mature miRNAs.

#### 4.2.2 Polyphenols

Polyphenols constitute another important class of plant-derived bioactive compounds widely distributed in fruits, vegetables, tea, cocoa, and red wine. Several polyphenols exhibit potent antioxidant, anti-inflammatory, and metabolic regulatory activities that are mediated, at least in part, through alterations in miRNA expression. Curcumin and resveratrol have been shown to influence multiple miRNAs involved in oxidative stress, mitochondrial function, inflammation, and glucose metabolism, including those associated with TXNIP regulation (66). Although direct phytochemical–miRNA interactions remain to be demonstrated experimentally, these compounds may indirectly influence RNA regulatory networks through modulation of signaling pathways, chromatin remodeling, RNA-processing enzymes, or, potentially, through allosteric interactions with structurally accessible RNA motifs. These proposed mechanisms warrant further investigation using integrated computational and structural biology approaches.

#### 4.2.3 Alkaloids

Alkaloids are structurally diverse nitrogen-containing natural products with well-established pharmacological activities. Several alkaloids, including berberine and caffeine, have been reported to regulate miRNA expression in pathways associated with inflammation, oxidative stress, and metabolic homeostasis (72). Their biological effects are believed to arise primarily through modulation of intracellular signaling cascades and transcriptional networks rather than direct interaction with mature miRNAs. Consequently, alkaloids represent promising candidates for indirectly regulating TXNIP-associated miRNA pathways and merit further investigation as potential modulators of RNA-mediated therapeutic networks.

### 4.3 Expression-specific mechanistic insights into key phytochemicals and their miRNA interactions

Current evidence indicates that individual phytochemicals regulate distinct sets of miRNAs through multiple interconnected molecular pathways, including transcriptional regulation, epigenetic remodeling, oxidative stress responses, and modulation of RNA-processing machinery. Although several phytochemicals have been associated with altered expression of TXNIP-regulating miRNAs, the precise molecular mechanisms responsible for these effects remain incompletely understood. **Table 1** has a summary of the principal phytochemicals discussed in this review, their reported miRNA targets, and the corresponding biological effects relevant to TXNIP regulation.

**Table 1:** Summary of phytochemical-miRNA interaction effects.

Phytochemical	Target miRNA	Effect on TXNIP	Biological outcome	References
Curcumin	miR-148b	Downregulates TXNIP	Reduces oxidative stress, improves metabolic balance	(72, 73)
Quercetin	miR-17-5p	Inhibits TXNIP translation	Lowers inflammation, enhances insulin sensitivity	(74, 75)
Resveratrol	miR-33a/b	Decreases TXNIP expression	Enhances mitochondrial function, reduces lipid accumulation	(76, 77)

#### 4.3.1 Curcumin

Curcumin has been reported to enhance the expression of miR-148b, a miRNA implicated in post-transcriptional regulation of TXNIP, thereby contributing to attenuation of oxidative stress and improved metabolic homeostasis (73). In addition, curcumin influences multiple signaling pathways involved in inflammation, redox balance, and epigenetic regulation that may collectively contribute to altered miRNA expression profiles (72). Although computational studies have suggested that RNA–ligand interactions may occur in structurally accessible regions of RNA molecules, direct interaction between curcumin and mature TXNIP-regulating miRNAs has not yet been experimentally demonstrated. Consequently, any proposed RNA structural modulation by curcumin should currently be regarded as a mechanistic hypothesis requiring further investigation.

#### 4.3.2 Quercetin

Quercetin has been shown to upregulate miR-17-5p, resulting in reduced TXNIP expression and subsequent attenuation of oxidative stress, inflammation, and insulin resistance (74). Beyond its effects on miRNA expression, quercetin improves endothelial function and cellular redox balance through multiple signaling pathways associated with TXNIP inhibition (75). These observations suggest that quercetin may exert coordinated regulatory effects on both transcriptional signaling and RNA-mediated gene regulation, although the precise molecular interactions linking these processes remain to be elucidated.

#### 4.3.3 Resveratrol

Resveratrol regulates miR-33a/b and other metabolic miRNAs associated with TXNIP expression, contributing to improved mitochondrial function, lipid homeostasis, and glucose metabolism (76). Activation of the SIRT1 signaling pathway and modulation of oxidative stress responses are believed to contribute substantially to these regulatory effects (77). While resveratrol has been proposed as a potential modulator of RNA regulatory networks, direct structural interactions between resveratrol and TXNIP-associated miRNAs remain hypothetical and await validation through integrated computational, structural, and biochemical studies (78).

### 4.4 Context-dependent miRNA regulation and potential off-target effects

The regulatory relationship between phytochemicals and miRNAs is highly context-dependent and is influenced by cellular environment, disease state, genetic variability, and RNA structural dynamics. Consequently, the biological effects of phytochemical-mediated modulation of TXNIP cannot be generalized across tissues or disease conditions. Instead, therapeutic outcomes are determined by a complex interplay among miRNA expression profiles, RNA-processing efficiency, intracellular signaling pathways, and individual genetic variation. Moreover, because many phytochemicals exhibit pleiotropic biological activities, they frequently influence multiple miRNAs and interconnected signaling networks beyond the intended therapeutic target, thereby increasing the possibility of off-target effects (79). Additional sources of variability include differences in absorption, metabolism, tissue distribution, and clearance, which may substantially alter pharmacological responses among individuals. Therefore, successful clinical translation of RNA-guided phytochemical therapeutics will require careful consideration of these context-dependent factors, together with comprehensive computational, structural, and experimental validation. The principal biological variables influencing phytochemical-miRNA interactions are summarized in **Table 2**.

**Table 2:** A summary of the principal biological variables influencing phytochemical-miRNA interactions.

S/N	Theme	Key subthemes	Description	References
a.	Influence of Cellular Environment and Disease State	Cellular environment, disease progression	miRNA expression varies with cell type, metabolic state, and disease stage, affecting phytochemical efficacy. In diabetes, oxidative stress alters miRNA processing; in cancer, miRNAs may act as oncogenes or tumor suppressors depending on context; in neurodegeneration, RNA-binding protein dysregulation alters miRNA–target interactions (e.g., TXNIP).	(66, 72)
b.	Genetic Variations in miRNA-Phytochemical Interactions	SNPs, miRNA seed region variation, inter-individual variability	SNPs in miRNA binding sites or seed regions can alter binding efficiency, leading to variability in phytochemical-mediated gene regulation, particularly TXNIP suppression.	(66, 72)
c.	Potential Off-Target Effects of Phytochemicals	Non-specific modulation, pathway cross-reactivity, pharmacokinetics	Phytochemicals can modulate multiple miRNAs unintentionally to affect unrelated pathways such as immunity, metabolism, or apoptosis. Variability in absorption and metabolism further influences safety and efficacy.	(66, 72)
d.	Cellular Environment Effects on miRNA–Phytochemical Interaction	Cell type specificity, metabolic state, oxidative stress	miRNA expression is cell-specific; hepatocytes vs β-cells show distinct regulatory profiles. High glucose and oxidative stress impair miRNA-mediated suppression of TXNIP, reducing phytochemical effectiveness.	(73, 75, 79)
e.	Disease State Influence on miRNA Dynamics	Diabetes, cancer, neurodegeneration	Disease states alter miRNA biogenesis and function. In diabetes, TXNIP overexpression and inflammation impair miRNA activity; in cancer, miRNA roles are context-dependent; in Alzheimer’s disease, RNA-binding protein dysfunction affects miRNA stability.	(73, 75, 79)
f.	Genetic Variations Affecting miRNA Regulation	SNPs, miRNA machinery mutations	SNPs in miRNA binding sites and mutations in Dicer, Drosha, or Argonaute proteins can disrupt miRNA processing and TXNIP targeting, leading to inter-individual variability in response to phytochemicals.	(66, 72)
g.	Non-Specific miRNA Modulation (Side Effects)	Multi-miRNA targeting	Phytochemicals may alter multiple miRNAs beyond intended targets. For example, resveratrol modulates both miR-33a/b (TXNIP-related) and miR-21 (oncogenic pathway).	(79–81)
h.	Cross-Reactivity with Other Pathways	Off-target signaling, immune and metabolic disruption	Phytochemicals like curcumin activate pathways (such as Nrf2), which may interact with oxidative stress regulation independently of miRNA mechanisms, potentially causing unintended effects.	(82, 83)
i.	Pharmacokinetic and Toxicity Challenges	Bioavailability, metabolism, toxicity	Variability in absorption, metabolism, and excretion affects therapeutic consistency. Chronic exposure may suppress essential genes, potentially leading to organ toxicity or immune dysfunction.	(66, 72)

## 5 Computational methods for miRNA-phytochemical interaction analysis

**Figure 7:**
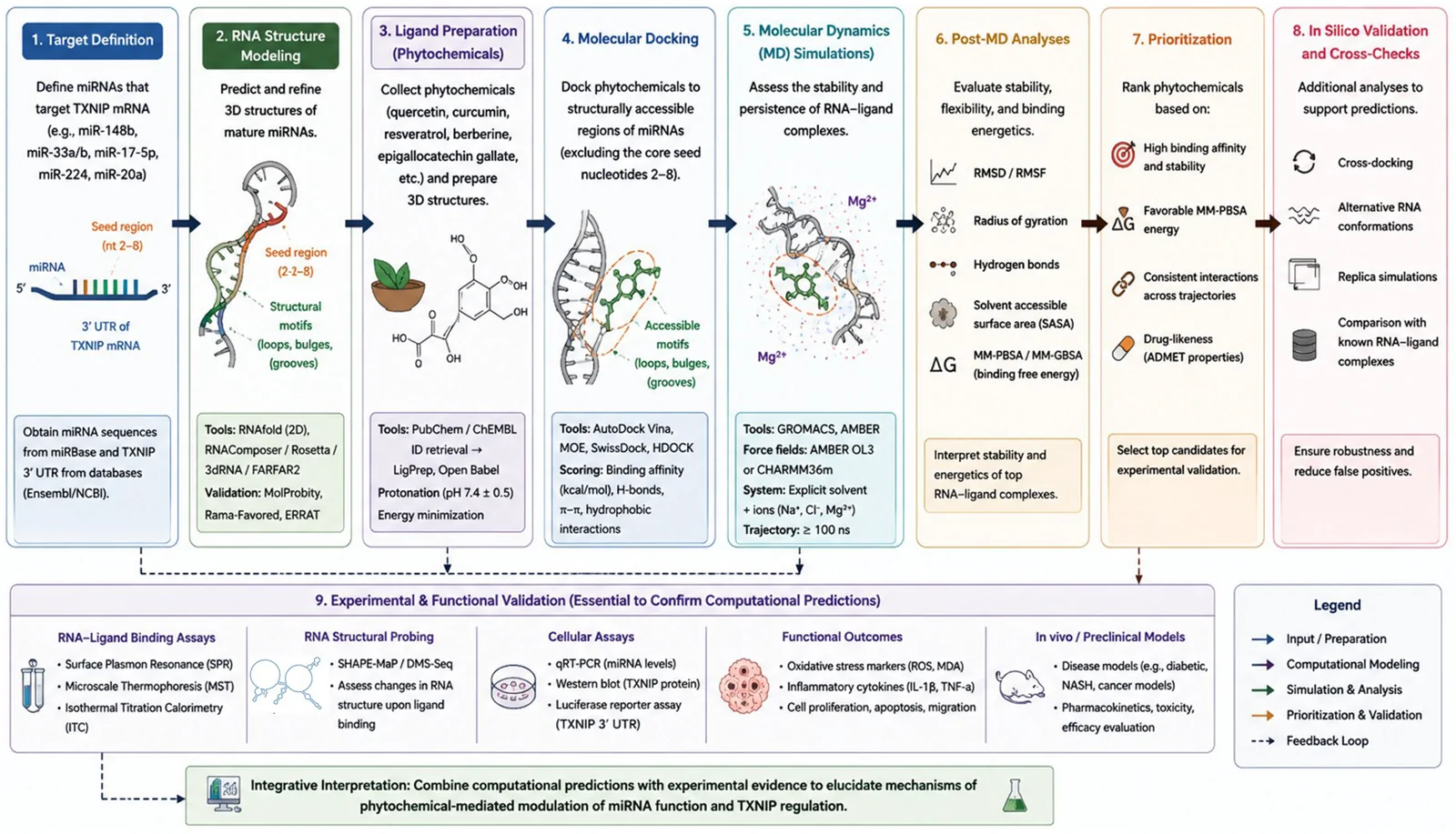
Computational workflow for investigating phytochemical-miRNA interactions targeting TXNIP. 3’ UTR: 3’ untranslated region; RSMD: root mean square deviation; RMSF: root mean square fluctuations; SASA: solvent accessible surface area; MM-PBSA: molecular mechanics Poisson-Boltzmann surface area; MM-GBSA: molecular mechanics generalized Born surface area; ADMET: adsorptsion, distribution, metabolism, excretion, and toxicity; DMS-seq: dimethyl sulfate sequence, ROS: reacting oxygen species; MDA: malondialdehyde; NASH: non-alchoholic steatohepatitis; SPR: surface plasmon resonance; MST: microscale thermophores; ITC: isothermal titration calorimetry; qRT-PCR: quantitative reverse transcription polymerase chain reaction.

Computational approaches have become indispensable for investigating RNA-small-molecule interactions and provide a rational framework for identifying phytochemicals capable of modulating miRNA function. Unlike proteins, mature miRNAs are highly flexible single-stranded RNA molecules that continuously sample multiple conformational states in solution. Consequently, computational analyses of miRNA-phytochemical interactions require methodologies that accurately capture RNA structural dynamics in addition to predicting binding affinity. An integrated workflow typically involves miRNA structure prediction, phytochemical preparation, molecular docking, molecular dynamics (MD) simulations, and binding free-energy calculations (30). Similar to Liu et al. (31), these complementary approaches can facilitate the identification and validation of potential RNA-binding phytochemicals for regulating TXNIP-associated miRNAs. The computational framework is presented in **Figure 7** and important components of the computational techniques are enumerated accordingly.

### 5.1 miRNA structure prediction

Accurate prediction of both the secondary and tertiary structures of miRNAs is an essential prerequisite for RNA-targeted molecular docking. Since mature miRNAs frequently lack experimentally determined structures, computational modelling is often required to generate biologically relevant three-dimensional conformations for docking studies (84, 85).

- Databases and prediction tools: Secondary structures may be predicted using RNAfold, whereas tertiary structures can be generated using platforms such as RNAComposer, Rosetta FARFAR2, SimRNA, or MC-Fold/MC-Sym. These tools reconstruct energetically favorable RNA conformations suitable for subsequent docking analyses (86).
- Three-dimensional structure acquisition: Whenever available, experimentally resolved RNA structures should be retrieved from repositories such as the Protein Data Bank (PDB), RCSB PDB, or the Nucleic Acid Database (NDB). In the absence of experimentally determined structures, homology modelling or *de novo* RNA structure prediction may be employed to generate plausible three-dimensional models before molecular docking (4).

### 5.2 Phytochemical preparation

Reliable molecular docking depends on careful ligand preparation to ensure chemically accurate and energetically stable phytochemical structures.

- Ligand retrieval: Candidate phytochemicals may be obtained from established chemical databases, including PubChem, ChEMBL, ZINC, NPASS, and the Traditional Chinese Medicine Systems Pharmacology Database (TCMSP). Molecular structures may also be generated using ChemDraw or ACD/ChemSketch before further optimization (87, 88).
- Molecular optimization: Ligands should undergo geometry optimization, hydrogen atom addition, protonation state assignment at physiological pH, charge calculation, and energy minimization before docking. Software such as UCSF Chimera, Open Babel, Avogadro, Gaussian, or RDKit can facilitate these preprocessing steps, thereby improving docking accuracy (4).

### 5.3 Molecular docking for miRNA–phytochemical interactions

RNA–ligand docking predicts potential binding poses and identifies structurally accessible RNA motifs capable of interacting with small molecules. Unlike conventional protein docking, RNA docking should account for the intrinsic flexibility of RNA molecules and the presence of multiple transient conformations.

- AutoDock4 and AutoDock Vina: These widely used open-source programs provide efficient docking of small molecules against RNA targets using empirical scoring functions and flexible ligand sampling. AutoDock is an open-access tool available at https://autodock.scripps.edu/download-autodock4/.
- Molecular operating environment (MOE): MOE offers advanced protocols for RNA–ligand docking, pharmacophore analysis, and interaction fingerprinting, enabling more detailed characterization of RNA–small-molecule complexes. However, MOE is limited by license for use, especially for commercial purposes, and is available at https://www.chemcomp.com/en/Products.htm.
- SwissDock and HDOCK: These web-based platforms provide complementary docking approaches and facilitate rapid screening of candidate phytochemicals against RNA structural models. Multiple docking platforms are often recommended because different scoring functions may yield complementary predictions of RNA-binding modes. They are available at https://www.swissdock.ch/ and http://hdock.phys.hust.edu.cn/, respectively.

Importantly, docking should primarily identify interactions with structurally accessible RNA motifs, including loops, bulges, grooves, junctions, or regions adjacent to the seed sequence, rather than assuming direct occupation of the canonical seed nucleotides required for Watson–Crick pairing.

### 5.4 Binding stability prediction as molecular dynamics validation

Because conventional molecular docking represents only a static snapshot of ligand binding, post-docking molecular dynamics (MD) simulations are essential for evaluating the stability and conformational evolution of RNA–ligand complexes under physiologically relevant conditions.

- RNA-specific molecular dynamics simulations: Mature miRNAs exhibit substantially greater conformational flexibility than globular proteins owing to their single-stranded architecture and highly charged phosphate backbone. Consequently, RNA simulations should employ force fields specifically optimized for nucleic acids, such as AMBER OL3, AMBER ff14SB/OL3, or CHARMM36m, which provide improved representation of RNA backbone torsions and base-stacking interactions. Explicit-solvent simulations should include physiologically relevant monovalent ions together with divalent magnesium ions (Mg^2+^) whenever appropriate, since Mg^2+^ plays a critical role in stabilizing RNA tertiary structure by partially neutralizing electrostatic repulsion between phosphate groups and maintaining biologically relevant conformations. To adequately capture RNA folding dynamics, ligand accommodation, and conformational fluctuations, production simulations of at least 100 ns are generally recommended, with longer trajectories providing greater confidence in the stability of RNA–ligand complexes. GROMACS is available at https://www.gromacs.org/, whereas AMBER is available at https://ambermd.org/.
- Binding free-energy calculations: Following MD equilibration, the thermodynamic stability of phytochemical–miRNA complexes can be estimated using methods such as MM-PBSA or MM-GBSA. These approaches provide more reliable estimates of binding energetics than docking scores alone by incorporating solvent effects and conformational flexibility across MD trajectories, thereby enabling more robust prioritization of candidate phytochemicals for subsequent experimental validation (89–91).

### 5.5 Molecular docking of phytochemical interactions with TXNIP-regulating miRNAs

A new class of druggable RNA molecules whose biological activity may be impacted by small-molecule ligands is represented by the mature structure of miRNAs. A useful tool for investigating how phytochemicals interact with physically accessible areas of TXNIP-regulating miRNAs and forecasting the possible effects of these interactions on RNA structure and function is molecular docking. According to the current report, small molecules are more likely to bind accessible structural motifs like internal loops, bulges, hairpin loops, junctions, grooves, or regions adjacent to the seed sequence rather than directly occupying the canonical seed region (nucleotides 2–8), which is necessary for Watson–Crick base pairing with complementary sequences in the 3′ untranslated region (3′UTR) of target mRNAs (92). The efficacy of miRNA-mediated suppression of TXNIP, target accessibility, Argonaute (AGO) loading, and miRNA stability may all be modulated by these interactions, which can cause local or long-range conformational changes. Insights for molecular dynamics simulations with potential for experimental validations are highlighted accordingly.

#### 5.5.1 Potential modulation of miRNA function

The biological consequences of phytochemical binding depend not only on binding affinity but also on the structural situation of the interaction. Following molecular docking, subsequent analyses should evaluate whether ligand binding promotes or perturbs biologically relevant RNA conformations. Such analysis can help to predict any of these two modulatory mechanisms.

- Allosteric enhancement of miRNA function: Phytochemicals that bind to structurally accessible regions may stabilize conformations that favour AGO loading, increase miRNA structural integrity, or improve accessibility of the seed region for complementary target recognition. Such allosteric modulation may strengthen miRNA-mediated repression of TXNIP without directly occupying the nucleotides responsible for Watson-Crick pairing (30).
- Functional disruption of miRNA activity: Conversely, ligand binding may induce conformational rearrangements that reduce seed accessibility, impair AGO recruitment, destabilize mature miRNA structures, or interfere with productive miRNA-mRNA duplex formation (31). These effects could diminish repression of TXNIP and consequently enhance oxidative stress, inflammatory signalling, or metabolic dysfunction.

#### 5.5.2 Identification of structurally accessible RNA binding sites

An important objective of RNA-ligand docking is the identification of structurally accessible binding sites capable of accommodating small molecules. These sites commonly include hairpin loops, internal bulges, multi-branch junctions, groove regions, and flexible nucleotides adjacent to the seed sequence rather than the canonical seed nucleotides themselves.

Identification of these structural hotspots enables investigators to:

- identify phytochemicals capable of stabilizing biologically relevant miRNA conformations that favour efficient TXNIP repression;
- recognize ligands that may induce unfavourable structural rearrangements capable of reducing miRNA regulatory efficiency; and
- prioritize candidate phytochemicals for subsequent molecular dynamics simulations and experimental validation.

#### 5.5.3 Assessment of binding affinity and RNA conformational dynamics

Although docking scores provide an initial estimate of RNA–ligand interaction strength, they represent static binding predictions and should therefore be interpreted alongside structural analyses.

- Binding affinity: Predicted binding affinity provides a preliminary indication of interaction strength between a phytochemical and structurally accessible regions of the miRNA. However, favourable docking scores alone do not necessarily imply biological activity.
- RNA conformational dynamics: Greater emphasis should be placed on evaluating ligand-induced conformational changes, including alterations in RNA flexibility, structural accessibility, tertiary folding, and the orientation of the seed region relative to the target mRNA. These dynamic structural changes may determine whether phytochemical binding ultimately enhances or attenuates TXNIP regulation.

#### 5.5.4 Comparative analysis of RNA-ligand binding modes

Comparative evaluation of multiple phytochemical docking poses provides insight into how different structural scaffolds may differentially modulate miRNA function.

- Identification of favourable allosteric modulators: Candidate phytochemicals that consistently stabilize biologically relevant RNA conformations or promote efficient target recognition may represent promising modulators of TXNIP-regulating miRNAs.
- Identification of potentially disruptive ligands: Conversely, compounds predicted to destabilize RNA structure, reduce seed accessibility, or interfere with AGO-mediated RNA silencing should be recognized as potentially undesirable modulators whose effects may compromise normal miRNA function.

Comparative analysis of binding modes, molecular dynamics trajectories, and binding free-energy calculations therefore provides a rational framework for prioritizing phytochemicals for experimental validation and the future development of RNA-targeted therapeutics.

### 5.6 Enhanced molecular docking analysis for phytochemicals and miRNA interactions

#### 5.6.1 Binding affinity data for key phytochemicals and miRNA interactions

Recent developments in computational chemistry and RNA structural biology have demonstrated that mature miRNAs may be recognised by small molecules, opening up new possibilities for RNA-targeted treatments. Natural compounds can create stable interactions with mature miRNAs through hydrogen bonding, π–π stacking, electrostatic interactions, and other non-covalent forces that affect RNA conformation and stability instead of just blocking RNA function, as shown by molecular docking and virtual screening techniques. Recent computational studies, for instance, have effectively docked natural chemicals to mature miRNAs and shown nucleotide-specific interaction patterns that may change the regulatory function and structural dynamics of miRNAs (92).

To date, no published study has directly assessed the molecular docking of major phytochemicals, such as curcumin, quercetin, and resveratrol, with the TXNIP-associated miRNAs mentioned in this review, even though these phytochemicals with antioxidant and anti-inflammatory properties have potential TXNIP-regulatory effects (93–96). Therefore, rather than being supported by experimental or computational data, the interactions suggested here should be viewed as a mechanistically informed hypothesis. We propose that these phytochemicals may interact with structurally accessible regions of mature miRNAs, such as internal bulges, stem-loop elements, terminal loops, or nucleotides adjacent to the seed region, resulting in local conformational changes that may modify Argonaute loading, target recognition, or miRNA stability. Compared to direct occupancy of the standard seed nucleotides (positions 2–8), which must continue to be accessible for Watson–Crick base pairing with complementary sequences in the 3′ untranslated region of TXNIP mRNA, such interactions are more physiologically realistic.

As a result, rather than using empirically verified binding affinities, **Table 3** offers a suggested computational methodology for assessing potential phytochemical-miRNA interactions pertinent to TXNIP regulation. To ascertain whether phytochemicals can allosterically modify the structure and function of TXNIP-regulating miRNAs and consequently affect downstream gene regulation (67), we suggest that future studies combining RNA molecular docking, molecular dynamics simulations, and experimental validation techniques like SHAPE-MaP, surface plasmon resonance, microscale thermophoresis, and Argonaute-loading assays will be crucial (67, 97).

**Table 3:** Representative phytochemicals and TXNIP-related miRNA with their associated biological effects.

Phytochemical	TXNIP-related miRNA	Proposed interaction	Current evidence	Future validation
Curcumin	miR-33a/b	Hypothesized RNA-ligand interaction affecting miRNA conformation and target recognition	Not yet directly evaluated	RNA docking and MD simulation
Quercetin	miR-148b	Hypothesized stabilization of mature miRNA structure	No direct evidence	Molecular docking and SHAPE validation
Resveratrol	miR-17-5p	Proposed modulation of RISC accessibility	Indirect evidence only (78)	Docking and AGO2 assays

#### 5.6.2 Limitations of docking studies

While molecular docking provides valuable predictions, several limitations must be addressed for a more comprehensive validation. Those challenges are highlighted as follows:

- Lack of Solvent and Flexibility Consideration: Docking studies assume rigid molecular structures. However, in biological systems, RNA and ligands exhibit conformational flexibility. Molecular dynamics (MD) simulations are necessary to refine docking predictions and account for dynamic movements.
- Absence of Cellular Situation: Computational docking does not account for miRNA processing, cellular localization, or competing endogenous RNA interactions, which can influence phytochemical efficacy.
- Experimental Validation Required: Predictions must be validated using biophysical techniques (e.g., surface plasmon resonance, isothermal titration calorimetry) to confirm direct interactions and functional assays (e.g., qRT-PCR, Western blotting) to assess TXNIP regulation.
- Bioavailability Considerations: Many phytochemicals have poor solubility and limited bioavailability. Further pharmacokinetic studies are needed to determine whether effective concentrations reach target tissues.

Therefore, these experimental validation strategies will provide robust evidence for the miRNA-mediated suppression of TXNIP by phytochemical interactions. Combining molecular, biochemical, and genetic approaches ensures a comprehensive evaluation, paving the way for translational applications in metabolic disease management. However, docking studies alone are insufficient. Molecular dynamics simulations and experimental validation must be integrated to enhance predictive accuracy and confirm functional relevance in biological systems.

## 6 Future perspectives on miRNA-based phytochemical therapeutics

Despite the growing body of computational, structural, and experimental evidence supporting the ability of phytochemicals to modulate miRNA regulatory networks, significant challenges remain before these findings can be translated into clinically applicable RNA-targeted therapeutics. Although computational approaches provide valuable hypotheses regarding potential phytochemical–miRNA interactions, they cannot independently establish biological function or therapeutic efficacy. Consequently, rigorous experimental validation, integration of RNA structural biology with computational modelling, and carefully designed preclinical studies are essential for confirming whether phytochemicals can modulate TXNIP-associated miRNAs under physiological conditions. Future progress will therefore depend on combining computational prediction, molecular dynamics simulations, structural validation, and functional cellular assays to establish mechanistic evidence supporting RNA-guided phytochemical therapeutics.

### 6.1 Experimental validation strategies

Computational analyses (30, 67, 97), including molecular docking and molecular dynamics simulations, generate mechanistic hypotheses regarding potential phytochemical interactions with TXNIP-regulating miRNAs. However, these predictions require experimental confirmation to determine whether the proposed interactions alter miRNA expression, stability, RNA-induced silencing complex (RISC) function, or downstream regulation of TXNIP (98). A combination of molecular, biochemical, and structural approaches can provide complementary evidence for validating these computational predictions.

#### 6.1.1 Quantitative real-time PCR for miRNA expression analysis

Quantitative real-time PCR (qRT-PCR) remains one of the most reliable approaches for determining whether phytochemical treatment alters the expression of TXNIP-associated miRNAs. Following treatment of cultured cells or experimental tissues with candidate phytochemicals such as quercetin, resveratrol, or berberine, total RNA may be isolated and reverse-transcribed prior to amplification using miRNA-specific primers, as described by Forero et al. (99). Changes in miRNA abundance can then be correlated with alterations in TXNIP expression and downstream cellular responses. Because phytochemicals may regulate miRNA expression indirectly through transcriptional, epigenetic, or post-transcriptional mechanisms, qRT-PCR provides an important first step in validating their influence on RNA regulatory networks.

#### 6.1.2 Western blotting for TXNIP protein expression analysis

As described by Nagaraj et al. (100), Western blotting can provide a direct functional readout of whether phytochemical-induced modulation of miRNA activity ultimately influences TXNIP protein expression. Following phytochemical treatment, proteins are extracted, separated by SDS-PAGE, and immunoblotted using antibodies specific for TXNIP. A reduction in TXNIP protein levels, accompanied by corresponding changes in regulatory miRNA expression, would support the hypothesis that phytochemicals modulate RNA-mediated repression of TXNIP. When interpreted alongside transcriptomic analyses, Western blotting can establish an important functional endpoint linking computational predictions with biological outcomes.

#### 6.1.3 Luciferase reporter assay for seed region interaction validation

The luciferase reporter assay described by Jin et al. (101) remains one of the most informative approaches for evaluating whether phytochemical treatment influences miRNA-mediated regulation of TXNIP. In this assay, cells are co-transfected with luciferase reporter constructs containing either wild-type or mutated TXNIP 3′ untranslated region (3′UTR) sequences together with the corresponding miRNA mimics or inhibitors. Candidate phytochemicals are subsequently administered, and luciferase activity is quantified. Reduced luciferase activity in cells containing the wild-type reporter, but not the mutant construct, indicates enhanced miRNA-dependent repression of TXNIP (101). Importantly, this assay evaluates the functional consequences of phytochemical-mediated modulation of miRNA activity rather than demonstrating direct binding of phytochemicals to the miRNA seed region. Consequently, luciferase assays provide robust evidence linking computational predictions to biologically relevant regulation of TXNIP expression.

When combined, luciferase reporter assays, Western blotting, and qRT-PCR offer a complementary experimental framework for verifying computational theories on phytochemical modulation of TXNIP-associated miRNAs. When combined with new RNA structural methods, such as surface plasmon resonance, microscale thermophoresis, Argonaute-loading assays, and selective 2′-hydroxyl acylation analysed by primer extension and mutational profiling (SHAPE-MaP), these methods can offer more convincing proof of RNA–ligand interactions and their functional implications. To convert computational predictions into clinically applicable RNA-guided phytochemical treatments, such interdisciplinary validation techniques will be crucial.

### 6.2 Potential for clinical translation and drug development

The capacity of phytochemicals to alter miRNA-mediated control of TXNIP provides a viable path for creating novel RNA-targeted treatments for cancer, metabolic disorders, and other chronic illnesses. Nevertheless, transferring this conceptual framework into clinical practice entails overcoming significant scientific and translational hurdles. These include establishing definitive mechanistic evidence for phytochemical-RNA interactions, improving the pharmacokinetic properties of candidate compounds, developing efficient RNA-targeted delivery systems, and validating therapeutic efficacy through rigors preclinical and clinical investigations. Integrating computational modelling, structural biology, pharmacology, and precision medicine will be vital for bringing phytochemical-based regulation of TXNIP-regulating miRNAs from hypothesis to clinical application (67).

#### 6.2.1 Phytochemical-derived miRNA therapeutics

Phytochemicals provide an intriguing supply of structurally varied small compounds that may function as modulators of RNA regulatory networks. Rather of operating only as traditional antioxidants or signalling modulators, some phytochemicals may alter miRNA expression, maturation, stability, or RNA structure dynamics, hence indirectly modulating TXNIP expression. Therefore, future therapeutic development should concentrate on finding phytochemicals with proven RNA-binding or RNA-modulatory qualities while enhancing their pharmacokinetic performance using formulation techniques like liposomes, nanoparticles, polymeric carriers, and other targeted delivery platforms. In parallel, structure-guided optimisation of naturally occurring phytochemicals may assist the synthesis of synthetic analogues with increased bioavailability, greater target selectivity, and enhanced power to influence disease-associated miRNA regulatory circuits (67, 98). These methods might eventually connect research on natural products with the development of next-generation RNA-targeted drugs.

#### 6.2.2 Precision medicine and RNA-guided therapeutic strategies

Because miRNA expression patterns differ significantly between persons, tissues, and disease states, precision medicine is anticipated to be crucial to the clinical translation of phytochemical-based RNA therapies. Patients who are most likely to benefit from phytochemical therapies targeting TXNIP-associated regulatory networks may be stratified by thorough molecular profiling of patient-specific miRNA signatures. Additionally, improvements in RNA delivery methods, such as extracellular vesicle-based delivery systems, tissue-specific nanoparticles, and ligand-directed carriers, may enhance treatment selectivity while reducing systemic exposure and off-target consequences. Integrating transcriptomic, epigenomic, and pharmacogenomic information with computational prediction of RNA–ligand interactions could facilitate individualised therapeutic strategies that maximise efficacy while reducing toxicity, thereby supporting the broader implementation of precision RNA medicine (28, 29).

#### 6.2.3 Preclinical and clinical trials

Robust preclinical validation remains indispensable before phytochemical-mediated modulation of TXNIP-regulating miRNAs can be translated into clinical practice. Experimental studies should combine computational predictions with molecular, biochemical, and structural validation to confirm the proposed mechanisms of RNA modulation. Disease-relevant cellular systems and animal models, including models of diabetes, MAFLD, neurodegeneration, and cancer characterized by dysregulated TXNIP expression, will be essential for evaluating therapeutic efficacy, pharmacokinetics, pharmacodynamics, and long-term safety (32, 33). Following successful preclinical validation, well-designed Phase I–III clinical trials should assess safety, optimal dosing, biomarker-guided patient selection, and therapeutic efficacy. Importantly, future clinical investigations should incorporate miRNA expression profiles, TXNIP biomarkers, and other molecular endpoints to establish target engagement and enable precision-guided therapeutic development (35). Such multidisciplinary validation will be essential before RNA-guided phytochemical therapeutics can become clinically applicable for managing chronic metabolic and oncologic diseases.

### 6.3 Implications of miRNA-phytochemical interactions for cancer evolution, diagnostics, and RNA therapeutics

As the global knowledge of RNA biology grows, the idea that cancer is only caused by genetic mutations has changed to the concept in which dynamic regulatory RNA networks play a significant role. Among them, microRNAs (miRNAs) have become important predictors of cancer development, progression, metastasis, and response to treatment (6, 10, 64, 85). Phytochemicals’ ability to alter miRNA activity has provided promising insight into RNA-guided therapeutic intervention through regulation of TXNIP, a critical molecular node linking oxidative stress, inflammation, metabolism, and tumour biology (18, 19, 56). Consistently, RNA-centered treatments have emerged as a new area of precision medicine due to the increasing convergence of regulatory RNAs, phytochemicals, and computational drug development (5, 102).

It is becoming increasingly acknowledged that TXNIP is a context-dependent regulator in cancer. TXNIP’s function as a tumour suppressor is supported by the fact that its expression is markedly decreased in a variety of cancers, including hepatocellular carcinoma, breast cancer, pancreatic cancer, colorectal cancer, and prostate cancer (18, 19, 32, 43). Mechanistically, TXNIP inhibits metabolic reprogramming, promotes oxidative stress-mediated apoptosis, negatively regulates glucose uptake, and opposes pro-survival pathways like PI3K/AKT, HIF-1α, and NF-κB signalling (18, 40, 49, 100). Cancer cells can maintain aerobic glycolysis, avoid apoptosis, and adjust to hypoxic tumour microenvironments when TXNIP expression is lost, which accelerates the growth of tumours (19, 32). Additionally, the TXNIP-HIF1α signalling axis has been directly linked to cancer metastasis, underscoring TXNIP’s significance as a regulator of cancer aggressiveness and evolution (65).

Many cancer-associated miRNAs closely control TXNIP expression and function. Oncogenic miRNAs can either directly or indirectly inhibit TXNIP signalling to promote tumour development and survival, according to new research. For instance, in breast cancer, miR-373 stimulates the epithelial-to-mesenchymal transition and metastasis via the miR-373–TXNIP–HIF1α–TWIST signalling axis (65). Similarly, miR-25-5p negatively controls TXNIP expression in inflammatory illness models (35), whereas miR-17-5p reduces TXNIP expression and affects redox-sensitive inflammatory pathways (52). Further evidence points to complex reciprocal regulation of TXNIP and miRNAs, as demonstrated by TXNIP-mediated modulation of the signalling pathways for miR-204 and miR-33a (53, 54). All of these results point to the miRNA-TXNIP axis as a crucial regulatory hub that integrates inflammation, cellular metabolism, oxidative stress responses, and carcinogenic transformation (56, 73).

Phytochemicals offer a unique opportunity to therapeutically modulate this regulatory network because of their capacity to influence both oncogenic and tumor-suppressive miRNAs. Natural substances, including curcumin, resveratrol, quercetin, genistein, epigallocatechin gallate, and other flavonoids, control miRNA synthesis, maturation, and target interactions, according to an increasing amount of research (7, 16, 72). According to reports, curcumin suppresses oncogenic miRNAs implicated in cancer growth and metastasis while restoring tumor-suppressive miRNAs (82, 83). In addition to modulating miRNAs linked to tumor-suppressor pathways (79, 80), resveratrol directly interacts with miR-33a and miR-122 and modifies their expression levels in hepatic cells (66). In a similar vein, genistein contributes to its anticancer properties by suppressing carcinogenic miR-155 (67). The capacity of flavonoids and plant-derived bioactive compounds to alter miRNA expression in glioblastoma, pancreatic cancer, breast cancer, and other cancers has been further demonstrated by recent research (68, 71, 92, 98). Through these interactions, phytochemicals may improve treatment results and slow the spread of cancer by indirectly restoring TXNIP function or rebalancing dysregulated redox signalling networks.

MiRNAs have become attractive circulating RNA biomarkers for cancer diagnosis, prognosis, and therapy monitoring in addition to their therapeutic uses. Because circulating miRNAs are shielded by extracellular vesicles, exosomes, and ribonucleoprotein complexes, they are exceptionally durable in blood and other bodily fluids (6). They are appealing candidates for minimally invasive liquid biopsy techniques due to their tissue-specific expression patterns and disease-associated dysregulation (85, 99). In cancer and metabolic illnesses, a number of miRNAs linked to oxidative stress, metabolism, and TXNIP-associated pathways have shown diagnostic and prognostic value (13, 55). As a result, miRNA profiling may help with patient stratification, treatment monitoring, early illness identification, and personalised therapy. Additionally, circulating miRNA profiles altered by phytochemicals may function as pharmacodynamic biomarkers for assessing the effectiveness of therapy.

RNA-targeted small molecules have emerged as a new therapeutic paradigm as a result of the identification of RNA molecules as druggable targets. RNA-targeted therapies seek to alter RNA structure, processing, stability, or function, in contrast to conventional medications that mainly target proteins (5). Recent advancements in computational modeling and RNA structural biology have enabled the identification of small compounds that can bind to mature miRNA complexes, precursor miRNAs, and structured RNA motifs (86, 103). Significantly, phytochemicals have shown promise as RNA-interacting compounds that can affect target identification and miRNA stability (66, 72). This idea fits within the larger context of RNA-guided drug discovery, in which naturally occurring compounds can act as scaffolds for the design of selective RNA-targeted therapeutics (5). The combination of molecular docking, molecular dynamics simulations, and experimental validation creates a robust platform for discovering phytochemicals that influence cancer-associated miRNAs and TXNIP-regulated pathways (4, 103).

In the future, it is anticipated that developments in AI will transform the process of discovering RNA-targeted drugs. Machine learning algorithms can combine transcriptomic, miRNA, proteomic, and interactome datasets to identify regulatory networks associated with diseases and predict therapeutic targets (102). The prediction of miRNA-mRNA interactions, RNA tertiary structures, and RNA-ligand binding sites has improved due to recent computational platforms, enhancing the accuracy of RNA-based therapeutic design (25, 28, 86). Moreover, there is a growing integration of AI-driven methods with network pharmacology, molecular docking, and molecular dynamics simulations to expedite phytochemical screening and enhance RNA-targeted drug development (95, 102, 104),. Research is beginning to investigate the possibility of using non-coding RNAs as drug candidates and molecular inhibitors, underscoring the growing field of RNA-based therapies (105). Such multidisciplinary approaches are closely aligned with the principles of precision oncology, where RNA signatures and RNA-targeted interventions are increasingly shaping the future of cancer diagnosis and treatment.

As a whole, these developments place interactions between miRNAs and phytochemicals at the crossroads of cancer evolution, molecular diagnostics, and RNA therapeutics. Gaining insight into the ways in which phytochemicals affect regulatory RNA networks—especially those that include TXNIP—could uncover new possibilities for biomarker identification, targeted therapies, and personalized medicine. With RNA-centered strategies transforming biomedical research, the combination of phytochemicals, computational biology, artificial intelligence, and RNA-targeted therapies presents a hopeful framework for addressing cancer and other chronic diseases in the precision medicine era (5, 72, 102).

### 6.4 Challenges and possible remedies for miRNA-Based phytochemical therapeutics

Although there has been considerable advancement in comprehending how phytochemicals influence miRNA expression for therapeutic purposes, various challenges and limitations persist, obstructing the effective creation and use of miRNA-based phytochemical treatments.

#### 6.4.1 Bioavailability and pharmacokinetics

One of the primary challenges in using phytochemicals therapeutically is their low bioavailability, rapid metabolism, and poor stability in physiological conditions. These factors reduce the efficacy of phytochemicals as viable therapeutic agents when administered orally or in other forms. Possible solutions to this issue consist of nanoformulations for encapsulating and safeguarding phytochemicals, chemical modifications aimed at enhancing their stability and absorption, and sustained-release formulations that enable prolonged activity and improved therapeutic results (29). These approaches aim to enhance the delivery and bioavailability of phytochemicals, making them more effective in targeting miRNA pathways.

#### 6.4.2 Off-target effects and specificity

Another major challenge is the inherent lack of specificity in phytochemical interactions. Since miRNAs usually control several genes, and phytochemicals can unintentionally influence numerous signaling pathways, this may result in off-target effects that produce unintended side effects. To address this issue, selective screening of phytochemicals through advanced technologies, such as high-throughput profiling of miRNA interactions, can help pinpoint specific interactions that modulate the desired miRNA pathways (106). Improved selectivity and precision in screening techniques will enhance the specificity of phytochemical-based therapies, reducing the risk of adverse effects and improving treatment outcomes.

#### 6.4.3 Lack of standardized experimental models

The field of phytochemical-miRNA research faces another significant hurdle in the lack of standardized experimental models. Variability in cell lines, animal models, and treatment protocols makes it challenging to compare results across different studies (107). This inconsistency can complicate the interpretation of data and hinder the advancement of phytochemical-based therapeutics. Standardized experimental frameworks and harmonized clinical protocols are needed to provide a consistent approach for evaluating the efficacy and safety of phytochemicals in miRNA modulation (97). Such standardization can allow for more reliable comparisons and more robust findings across studies.

#### 6.4.4 Complexity of miRNA-phytochemical interactions

The complexity of miRNA regulation adds another layer of difficulty to predicting the long-term effects of phytochemical interventions. miRNA functions are dynamic, and their effects can vary depending on the cellular environment, the stage of disease progression, and interactions with other molecular pathways (108). As a result, predicting the therapeutic potential and long-term impact of phytochemical-induced miRNA regulation is a complex task. To improve predictive accuracy, machine learning models and AI-driven miRNA analysis can be leveraged to create more sophisticated and reliable tools for forecasting the outcomes of phytochemical interventions (102). Therefore, these computational approaches can help to predict phytochemicals’ effects on miRNA targets for better-informed therapeutic strategies.

## 7 Conclusion

The emerging field of phytochemical-mediated modulation miRNA regulatory networks offers a promising frontier for the development of innovative therapeutic strategies against cancer, MAFLD, diabetes, cardiovascular diseases, neurodegenerative disorders, and other chronic conditions characterized by oxidative stress and inflammation. Current evidence supports the ability of phytochemicals to influence miRNA expression and function through transcriptional, epigenetic, redox-sensitive, and post-transcriptional regulatory mechanisms, thereby contributing to the modulation of key molecular targets such as TXNIP linked to oxidative stress and inflammation, which are pathophysiology of molecular diseases. In this narrative review, we further propose a mechanistically plausible framework in which phytochemicals may allosterically influence structurally accessible regions of mature miRNAs or associated RNA–protein complexes, potentially altering RNA conformation, stability, RISC loading, and target recognition without disrupting the canonical seed-region interactions required for Watson-Crick base pairing. Although advances in molecular docking, RNA structural prediction, and RNA-specific molecular dynamics simulations provide valuable tools for generating hypotheses regarding phytochemical-RNA interactions, these computational predictions require rigorous validation through complementary structural, biochemical, cellular, and *in vivo* investigations. Future studies integrating computational modelling with RNA structural biology, multi-omics technologies, and precision medicine approaches will be essential for establishing the molecular basis and therapeutic relevance of these proposed interactions. In general, this review provides a conceptual roadmap for future research and highlights the potential of RNA-guided phytochemical therapeutics as an emerging strategy for precision oncology and the management of metabolic and other chronic diseases.
